# Phage therapy administered noninvasively could be effective in thin tubes subject to episodic flow despite washout: a simulation study

**DOI:** 10.1088/1478-3975/ab2ea0

**Published:** 2019-07-22

**Authors:** Celia Blanco, Irene A Chen

**Affiliations:** 1 Department of Chemistry and Biochemistry 9510, University of California, Santa Barbara, CA 93106, United States of America; 2 Program in Biomolecular Sciences and Engineering, University of California, Santa Barbara, CA 93106, United States of America; 3 Department of Chemical and Biomolecular Engineering, University of California, Los Angeles, CA 90095, United States of America; 4 Author to whom any correspondence should be addressed.

**Keywords:** urinary tract infection, phage therapy, computational model

## Abstract

Bacteriophages (phages) have been proposed as candidates for the treatment of bacterial infections in light of emerging antibiotic-resistant microorganisms. Bacterial growth within thin tubes is a particular concern, such as in urinary tract infections and colonization of catheters. However, it is not clear whether phage administration to the urinary tract or in catheters could be effective in the context of flow to the outside (i.e. voiding or saline flush). Here, we adapt a previous model of phage infection to a thin tube geometry mimicking the spatial organization of the urinary tract, including bacterial motility and episodic flow during which phages are washed out of the system. We show that density-dependent dynamics permit propagation of the phage infection and that washout has little effect on the timing of bacterial clearance. In addition, instillation of phage at the bottom ~0.1 mm of the tract is effective in our computational model, suggesting that therapeutic phage introduced non-invasively could be efficacious in such situations.

## 1. Introduction

Bacterial infections represent a growing threat to human health as antibiotic resistance genes spread among pathogenic organisms. The main selective pressure driving this trend is the use of antibiotics itself, underscoring the importance of additional therapeutic options. Bacteriophages (phages) represent an orthogonal therapeutic strategy that has garnered increasing attention (reviewed elsewhere (Sulakvelidze *et al* 2001, Summers 2001, Lu and Koeris 2011, Rose *et al* 2014, Abedon *et al* 2017, Lin *et al* 2017)). Phages possess several potential advantages compared to antibiotics (Sulakvelidze *et al* 2001, Abedon *et al* 2011). Depending on the phage strain, phages can have high specificity for the targeted bacteria or a more broad-spectrum effect (Flores *et al* 2011). Phages also replicate exponentially, potentially reducing the required frequency of dose administration, and can, in principle, be evolved *in vitro* to overcome resistance. However, phages present multiple challenges to systemic administration, including rapid clearance and poor bioavailability to potential target organs (Ly-Chatain 2014). Therefore, at this point, most current interest in phage therapy focuses on treatment of externally accessible infections, such as burns (Merabishvili *et al* 2017, Jault *et al* 2019) or infections of the intestinal tract (Tetz and Tetz 2016, Lusiak-Szelachowska *et al* 2017). Among these are urinary tract infections (UTIs), which are among the most common hospital and community-acquired bacterial infections. In the USA alone, UTIs affect millions of people and result in hundreds of thousands of hospitalizations and 12 000 deaths each year (Flores-Mireles *et al* 2015, Waller *et al* 2018). The incidence of UTIs caused by antibiotic-resistant bacteria, including vancomycin-resistant enterococcus and multidrug-resistant uropathogenic *Escherichia coli*, is rising (Kallonen *et al* 2017) (e.g. >20% of infections are resistant to the first line antibiotic trimethoprimsulfamethoxazole (Waller *et al* 2018)).

While phages could be a promising tool for treating UTIs, current work has been limited to *in vitro* demonstrations or invasive administration. For example, phage isolates were tested on *E. coli* and *Klebsiella pneumoniae* strains isolated from the urine of patients with UTIs, showing high lytic activity against the strains *in vitro* (Zhang 2014, Sybesma *et al* 2016). In three mouse models of UTIs, including infection by uropathogenic *E. coli*, phage injected intraperitoneally was effective in decreasing bacterial titer and improving survival (Nishikawa *et al* 2008, Tothova *et al* 2011 Dufour *et al* 2016). A planned human trial proposes administration via suprapubic catheter (twice per day for seven days) (Leitner *et al* 2017). Despite the risks associated with invasive administration, these approaches are taken because of the very low bioavailability of orally administered phage (Bruttin and Brus sow 2005). A safer alternative would be highly desirable. Administration of phages to the lower urinary tract is less invasive, but a clear concern is episodic flow, which would flush phages from the system. However, phages are self-replicating entities, and as such, the dynamics of their populations are not always intuitive. For example, phages exhibit density-dependent behaviors leading to unforgiving time thresholds that determine whether inoculation of a dose is successful or unfruitful (Payne and Jansen 2000, Payne and Jansen 2001). Despite their great potential as antibacterial agents, this lack of understanding has been one issue hampering clinical applications of phage therapy.

Therefore, in this work we model the dynamics of phage applied to a thin tube colonized by bacteria, undergoing episodic flow. The model is relevant to UTIs as well as other environments of potential interest for phage application, such as colonization of catheters, intravenous lines, drains, or other tubing. We adapt a previous model of lytic phage infection by placement in a thin tube and including diffusion of microbes and an episode of flow, during which free phages are washed out of the system. The results from the simulations indicate that even if free phages are completely cleared from the system during the flow, complete elimination of bacteria is still possible if some bacteria were infected after phage inoculation. Thus, we suggest that a single dose of phage administration could be effective in clearing bacterial populations in thin tubes despite counter-acting flow.

## 2. Model

We adapted a previously proposed kinetic model of lytic bacteriophage infection (Payne and Jansen 2001). In this simple model, *u*(*t*) is the amount of uninfected bacterial cells (*U*), *y* (*t*) is the amount of cells infected by bacteriophage (*Y*), and *p* (*t*) the amount of free phage (*P*). Free phages infect cells with rate constant *b*. Uninfected and infected bacteria replicate with rate constant *a*. The lysis rate constant, *k*, represents the death of the infected host cell by lysis and the release of phages, where *L*_*b*_ is the burst size (i.e. number of particles released per burst). Free phages are inactivated or degraded in the milieu with rate constant *m*.

**Figure 1.**
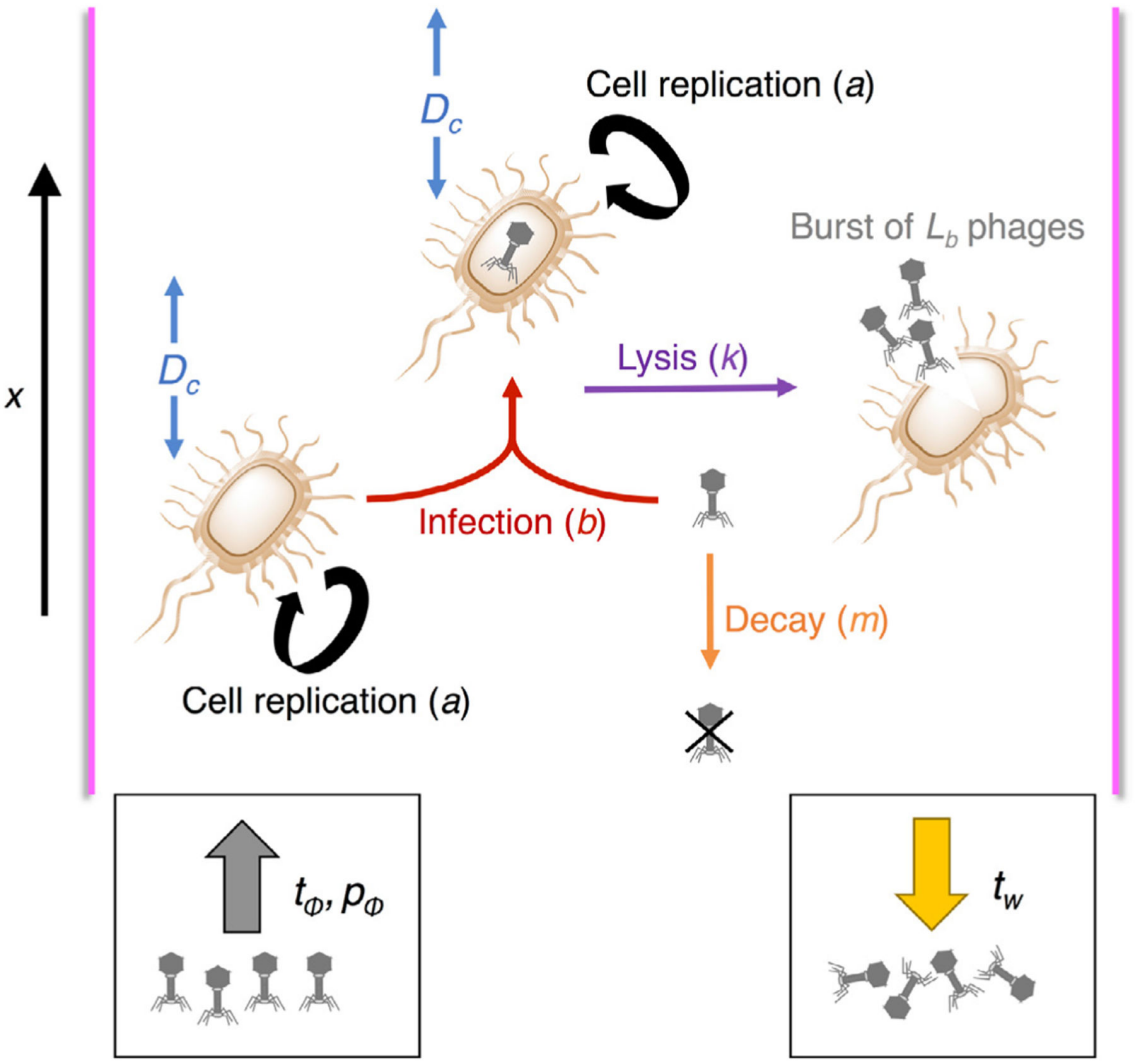
Schematic model for phage infection in a thin channel, including bacterial diffusion and phage washout.

We adapt the model to include a spatial coordinate *x*, simulating a long channel in which the radial dimension is considered to be much smaller than the longitudinal dimension, and growth of bacteria and phage is modeled along one dimension. Uninfected and infected bacteria diffuse with coefficient *D*_*c*_ to model bacterial motility (Berg 1993). Diffusion of free phages is neglected because physical diffusion of phage particles would be much slower than the spread of bacteria via swimming (Berg 1993). Before addition of phage, all cells are assumed to be uninfected and the phage concentration is zero. A bolus of therapeutic phage is introduced at time *t*_*Φ*_, with phage dose *p*_*Φ*_. At time *t*_*w*_, the system experiences washout (e.g. voiding), at which time all phages are eliminated (figure 1).

The system is described by the following equations (table 1), following the previous model (Payne and Jansen 2001) and Fick’s second law of diffusion. For *t* > 0, the system contains only uninfected cells, whose time evolution is described as follows:

(1) $$\frac{\partial u(t)}{\partial t}=au(t)-bp(t)u(t)+D_{c}\frac{\partial^{2}u(t)}{\partial x^{2}}.$$

Upon addition of phage, for *t* ≥ *t*_*ϕ*_, we also have infected cells and phage

(2) $$\frac{\partial y(t)}{\partial t}=ay(t)+bp(t)u(t)-ky(t)+D_{c}\frac{\partial^{2}y(t)}{\partial x^{2}}$$

(3) $$\frac{\partial p(t)}{\partial t}=kL_{b}y(t)-bp(t)u(t)-mp(t).$$

### 2.1. Methods

#### 2.1.1. Numerical solution to the model

The Crank–Nicolson method was used to solve the system numerically. The Crank–Nicolson method is a finite difference method used for numerical simulation of partial differential equations and is commonly applied to diffusion problems (Crank and Nicolson 1996). This method can be regarded as a combination of the forward Euler method and the backward Euler method. We constructed a 2D Crank–Nicolson grid, with *N* × *J* steps, where each time step and each space step has a fixed length. We aim to approximate the continuous functions *u* (*x*, *t*),*y*(*x*, *t*) and *p*(*x*, *t*) by their grid functions $u_{j}^{\left(n\right)}$, $y_{j}^{\left(n\right)}$ and $p_{j}^{\left(n\right)}$, where $u_{j}^{\left(n\right)}\approx u\left(x_{j},t_{n}\right)$, $y_{j}^{\left(n\right)}\approx y\left(x_{j},t_{n}\right)$ and $p_{j}^{\left(n\right)}\approx p\left(x_{j},t_{n}\right)$ at the *j*th spatial coordinate and *n*th time step.

For a total simulated time *T* and system of length *L*: *t*_*n*_ = *n*Δ*t* for *n* = 0, … ,*N −* 1, where $\Delta t=\frac{T}{N-1}$, and *x*_*j*_ = *j*Δx for *j* = 0, … , *J −* 1,where $\Delta x=\frac{L}{J-1}$.

The Crank–Nicolson equation is second-order in time:

$$\frac{z_{j}^{\left(n+1\right)}-z_{j}^{\left(n\right)}}{\Delta t}=\frac{D_{c}}{2{\left(\Delta x\right)}^{2}}(z_{j+1}^{\left(n\right)}-2z_{j}^{\left(n\right)}+z_{j-1}^{\left(n\right)}+z_{j+1}^{\left(n+1\right)}-2z_{j}^{\left(n+1\right)}+z_{j-1}^{\left(n+1\right)}),$$

where *z* = *u*, *y*, or *p*.

For every simulation, the total simulated time is *T* = 60 h (number of steps *N* = 6000) and the length of the system is *L* = 2 cm (number of steps *J* = 200). The length of the system is based on female urethra length values reported in the literature (19–45 mm) (Pomian *et al* 2018).

#### 2.1.2. Parameters of the model

Simulations of therapeutic responses were computed using biologically meaningful parameters (table 2) for bacterial replication rate constant (*a*), infection rate constant (*b*), lysis rate constant (*k*), and decay rate constant (*m*), from Payne and Jansen (2001). Burst size, bacterial diffusion, and washout time were considered constant across conditions. Bacterial diffusion terms were set based on values for motile *E. coli* (Berg 1993). Four sets of conditions were investigated. Conditions (a) and (b) represent rapid turnover systems, with faster bacterial replication, phage infection, lysis, and degradation compared to conditions (c) and (d). Condition (b) differs from (a) in having 10-fold smaller inoculum of phage (*p*_Φ_). The slower turnover conditions (c) and (d) receive a much smaller inoculum to probe the lower limit of *p*_Φ_ that could still reduce the bacterial population. Between the slow turnover conditions (c) and (d), the phage inoculum was applied later in d) to probe the importance of *t*_Φ_. For every case, initial conditions were *u*(0) = 1000 cells, *y* (0) = 0 cells and *p* (0) = 0 pfu (plaque-forming units); in other words, the systems began with no phage or infected bacteria. Bacteria were initially evenly distributed along the lowest 0.5% of *x* (i.e. the lowest 0.1 mm) to simulate an ascending infection.

## 3. Results

**Figure 2.**
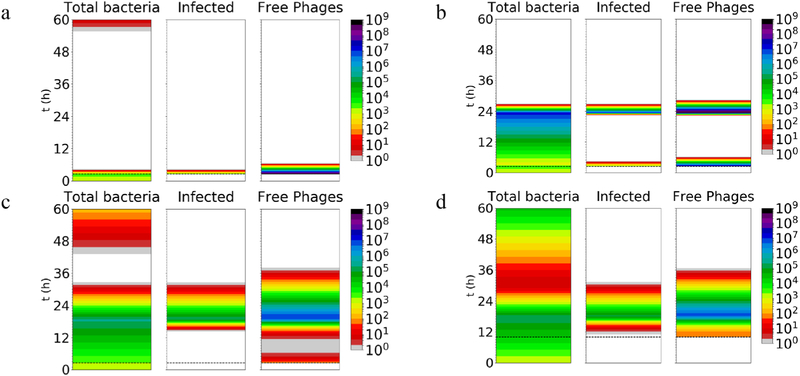
Amount of total bacteria, infected bacteria, and phage simulated for 60 h under conditions (a)–(d) (see table 2), with no bacterial diffusion or phage washout. The heat map indicates number of particles (cells or phages). The white color indicates amount < 1 cells or pfu. In a real situation, low populations (e.g. < 1) may represent eradication, such that regrowth afterwards would not be possible. The phage dose is applied at the time indicated by the dashed horizontal line. Note that there is no spatial dimension (i.e. well-mixed; *J* = 1).

As a basis for comparison, we first simulated the system described by the set of equations above (table 1), using the conditions indicated (table 2) (Payne and Jansen 2001) with no bacterial diffusion or washout (i.e. parameters identical to Payne and Jansen (2001), carried out for longer time). This scenario mimics a well-mixed environment. In condition (a), a large dose of phage eradicated the bacteria almost immediately (down to a population of < 1 cell) and essentially prevented bacterial replication (figure 2(a)). A smaller dose (condition (b)) also eventually cleared the bacteria, but only after a period of substantial bacterial growth (up to ~24 h; figure 2(b)). In the slow turnover systems, the bacteria are brought to low counts at around 24 h even by the low phage doses applied late into the infection (conditions (c) and (d); figure 2(c)). If the dose is applied late (condition (d)), it appears that the greater bacterial density leads to more efficient spread of the phage infection, such that the precise timing of phage inoculation does not substantially affect the time of bacterial clearance. In some cases, although the phage greatly reduces bacterial numbers initially, resulting in very low amounts (<10), the bacteria could potentially regrow from low numbers afterwards (figures 2(a), (c) and (d)); this effect was not previously observed in shorter simulations (Payne and Jansen 2001). Such regrowth might be problematic given the very low numbers of phages during this phase, which might lead to stochastic loss of the phage infection. However, potential regrowth of bacteria from such low numbers may or may not occur in real-life situations in which a functional immune system may efficiently clear very low numbers of bacteria.

**Figure 3.**
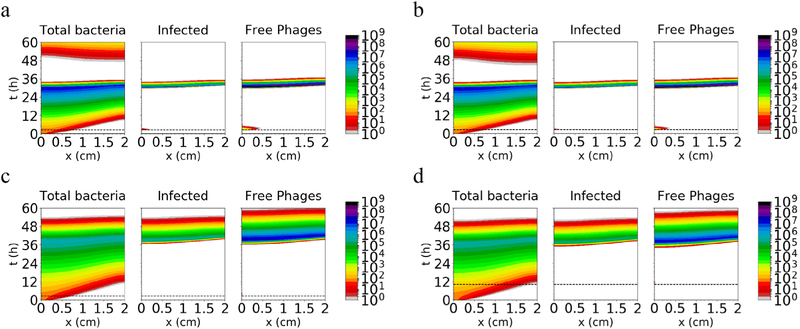
Amount of total bacteria, infected bacteria and free phages simulated for 60 h in a 2 cm tract, using the same conditions as in figure 2 and adding bacterial motility with *D*_*c*_ = 0.0144 cm^2^ h^−1^. There is no washout of phages in these scenarios. The phage dose is applied at the time indicated by the dashed horizontal line.

To evaluate the effect of spatial structure, we introduced diffusion of bacterial cells into the system, and used otherwise identical parameters for simulation. To model ascending infection, the bacteria were initially located in the lowest 0.5% of the tract (i.e. the lowest 0.1 mm), distributed evenly. Phages were also inoculated at the appropriate time (*t*_Φ_) at the lowest 0.1 mm of the tract. As shown in figure 3, the spatial structure delays eradication, particularly with the large inoculum, but bacteria are eventually reduced to low numbers from the entire tract in every case (at *t ~* 36 h for rapid-turnover conditions (a) and (b), and *t* ~ 48 h for slow-turnover conditions (c) and (d)). These results indicate that the phage infection can ‘outrun’ a motile bacterial population in a 1D channel.

**Figure 4.**
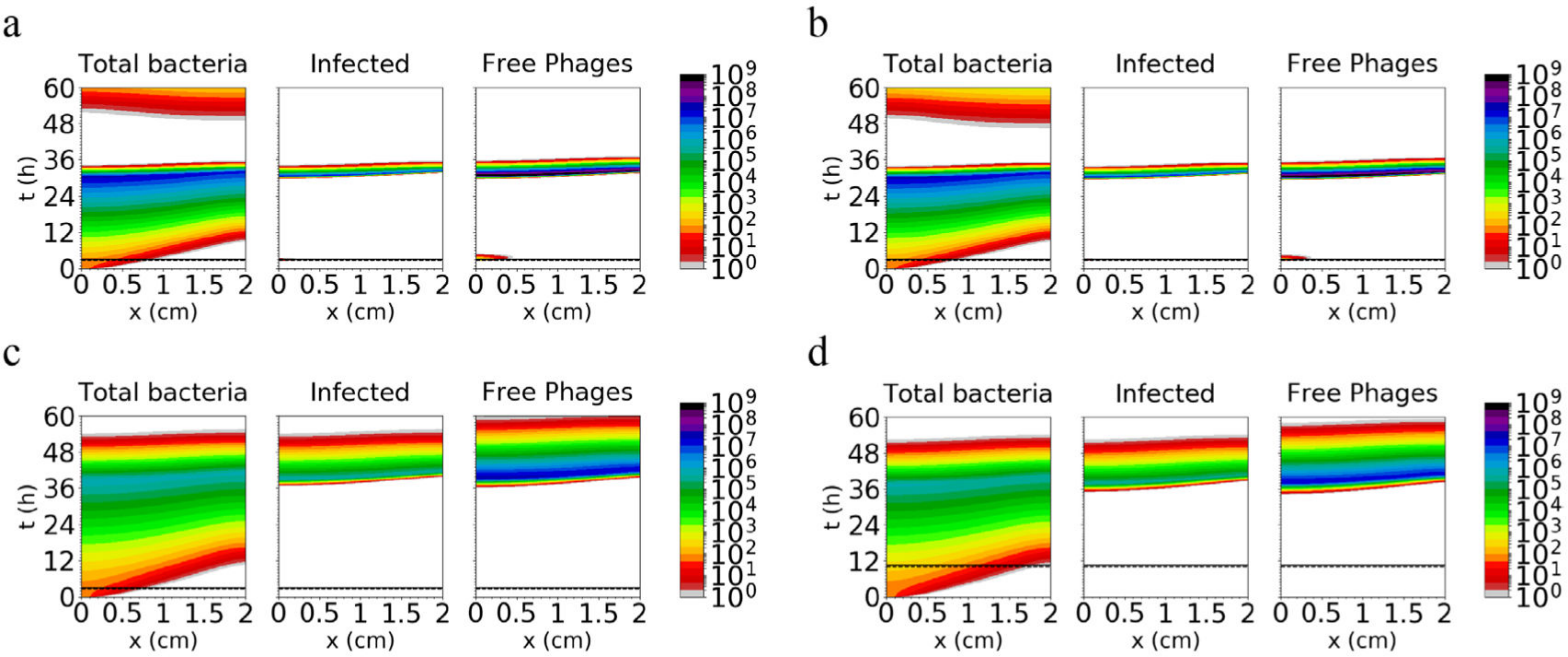
Amount of total bacteria, infected bacteria and free phages simulated for 60 h along a 2 cm tract, using the same conditions as figure 3 and including both bacterial motility and washout of phages. The phage dose is applied at the time indicated by the dashed horizontal line; washout occurs 30 min later, at the solid horizontal line.

To evaluate the effect of washout of phages, complete washout is introduced 0.5 h after inoculation of the phage for every case. At this time *t*_*w*_, free phages are eliminated from the system. With both inoculum conditions under rapid turnover (conditions (a) and (b); figures 4(a) and (b)), the bacteria are cleared at a time similar to the scenario without washout (*t* ~ 36 h), because the phage infection became established in the bacterial population before washout. These infected bacteria harbor the phage during washout and then lyse, releasing new free phages into the system that propagate further and eventually eliminate the bacteria in the tract. Qualitatively similar results are obtained in both slow-turnover conditions ((c) and (d); figures 4(c) and (d)), compared to the scenario without washout, in which the phages are able to establish an infection even with a low inoculum (bacterial population dropping to low numbers at ~48 h). Wash-out, even if occurring a relatively short time (0.5 h) after phage inoculation, therefore has a relatively small effect on the ability of the phage to reduce the bacterial population.

As expected, similar results are obtained when washout occurs later, corresponding to washout 3.5, 9.5, or 21.5 h after application of the phage dose (*t*_*w*_ = 6, 12 or 24 h when *t*_Φ_ = 2.5 h, or *t*_*w*_ = 13.5, 19.5 or 31.5 h when *t*_*Φ*_ = 10 h) (figures S1–S3) (stacks.iop.org/PhysBio/16/054001/mmedia).

We also examined whether the results were sensitive to the assumption that uninfected and infected cells replicate with the same rate constant (*a*). Since uninfected cells may replicate more slowly, we carried out simulations with the same parameters as in figure 4, except with uninfected cells replicating with rate constant *a*/2 (figure S4) or *a*/10 (figure S5). The results are not substantially affected by these adjustments, possibly because replication of infected cells is relatively limited due to lytic burst.

## 4. Discussion

In principle, phage therapy to treat UTIs or other bacterial infections in environments subject to episodic flow might seem untenable due to the washout of phages. However, phages are self-replicating entities whose antibacterial activity depends on bacterial concentration, leading to unpredictable effects (Payne and Jansen 2000, 2001). In this computational study, we find that these effects could prevent complete elimination of phages and thus facilitate phage therapy despite washout. While spatial structure, bacterial motility and washout changed the detailed outcome of the simulations compared to the results in the absence of these terms, particularly by delaying bacterial clearance, there was little qualitative change in the efficacy of the phage treatment. Although bacteria move at random away from the applied phage and free phages are washed out at a specific time, the phage treatment persists due to the production of a reservoir of infected cells, which leads to secondary infection as new phages are released by lytic burst. The size of the phage inoculum did not appear to be critical; a bigger inoculum could clear the bacterial population more quickly, but small inocula were effective as well, presumably owing to the exponential amplification dynamics of the phage. Also, in the simulation scenario explored here, both bacteria and phages were introduced at the lowest 0.1 mm of the tract, a position that would be easily accessible for a potential treatment. The ability of the phages to clear the bacterial population when applied in this position indicates that intravesicular instillation of phages to treat UTIs (Sybesma *et al* 2016) may be unnecessarily invasive. Phage applied noninvasively may be a promising avenue to counter bacterial infections despite concerns about the unique characteristics of the urinary tract and similar geometries.

The need for infected cells to propagate the phage in this context supports the theoretical concern that antibiotics and bacteriophages may not always interact synergistically, as antibiotic-mediated killing of bacteria would reduce the population of infected cells (Payne *et al* 2000, Payne and Jansen 2003). Antibiotics and phages have been observed to interact synergistically in experimental models (Knezevic *et al* 2013, Chaudhry *et al* 2017, Oechslin *et al* 2017, Chang *et al* 2019), and further studies of such interactions would be of interest.

It should be noted that application of this model to specific systems would require knowledge (i.e. experimental measurement) of the system parameters. In the conditions explored here, we assumed that cell motility is similar for infected versus non-infected cells and found that bacterial motility was insufficient to ‘outrun’ the phage infection. This situation may or may not hold for specific phages and bacteria; if phage infection inhibits motility, progress of the phage infection through the bacterial population may be impeded, delaying resolution of the bacterial infection. We also assumed that bacterial cells are not washed out (e.g. due to binding to the epithelium) while phages are entirely eliminated during voiding. In terms of persistence of the bacterial infection, this assumption represents a ‘worst-case’ scenario, and thus a relatively stringent test of washout. However, phages may become embedded in the mucosa, avoiding washout (Barr *et al* 2013, 2015), and voiding appears to play a role in reducing recurrent UTIs, presumably by decreasing bacterial load (Minardi *et al* 2011). Both effects would be expected to favor resolution of the infection, although simulation studies would be useful for validation. Finally, we did not consider evolution (i.e. emergence of phage-resistant bacteria and subsequent viral evolution (Meyer *et al* 2010, Kashiwagi and Yomo 2011)), or the role of the human immune system, although we presume that the immune system would be able to clear very low numbers of bacteria. Nevertheless, this computational model might be useful for exploring the potential of phage therapy of bacterial colonization in other tube-like contexts subject to flow, such as catheters, which are also an important nidus of infection. A lack of understanding of the pharmacokinetics of replicating phages is one of the major translational difficulties facing phages as a possible antibiotic therapy (Hesse and Adhya 2019). Thus, such models represent a useful route to explore scenarios for phage therapy and aid prediction of potential outcomes.

## Supplementary Material

Supplement

## Figures and Tables

**Table 1.** Reactions and parameters of the model.

Cell replication	$U\overset{a}{\to }2U$
	$Y\overset{a}{\to }2Y$
Phage infection	$P+U\overset{b}{\to }Y$
Lysis of host cell	$Y\overset{k}{\to }L_{b}P$
Degradation of phage	$P\overset{m}{\to }\emptyset$
Bacterial motility	*D*_*c*_
Phage application	At time *t*_Φ_, phage dose *p*_Φ_, is applied
Washout	Time *t*_*w*_

**Table 2.** Parameter values studied here for four conditions (a)–(d). The value of *p*_Φ_ is given as the number of phages applied to the lowest 0.1 mm of the tube. For parameter *b*, the term particle refers to either cells (equations (1) and (2)) or pfu (equation (3)).

Parameter	(a)	(b)	(c)	(d)
*a*	0.5 h^−1^		0.3 h^−1^	
*b*	10^−7^ particle^−1^ h^−1^		10^−6^ particle^−1^ h^−1^	
*k*	5 h^−1^		1.2 h^−1^	
*L*_*b*_	100			
*m*	5 h^−1^		1.8 h^−1^	
*t*_Φ_		2.5 h		10 h
*p*_Φ_	10^9^ pfu	10^8^ pfu		100 pfu
*D*_*c*_	1.44 10^−2^ cm^2^ h^−1^ (when applicable)			
*t*_*w*_	*t*_Φ_ + 0.5 h (when applicable)			
