# Supplementary material for: Phage therapy administered noninvasively could be effective in thin tubes subject to episodic flow despite washout: a simulation study
Source: Phys Biol. Author manuscript; Available in PMC 2019 Oct 1. (PMC6771420; doi:10.1088/1478-3975/ab2ea0)
Supplement: Supplement [file NIHMS1051836-supplement-Supplement.pdf]

Supplementary Information:

Phage therapy administered noninvasively could be effective in thin tubes subject to episodic flow despite washout: a simulation study

Celia Blanco and Irene A. Chen

Supplementary Figures S1-S5

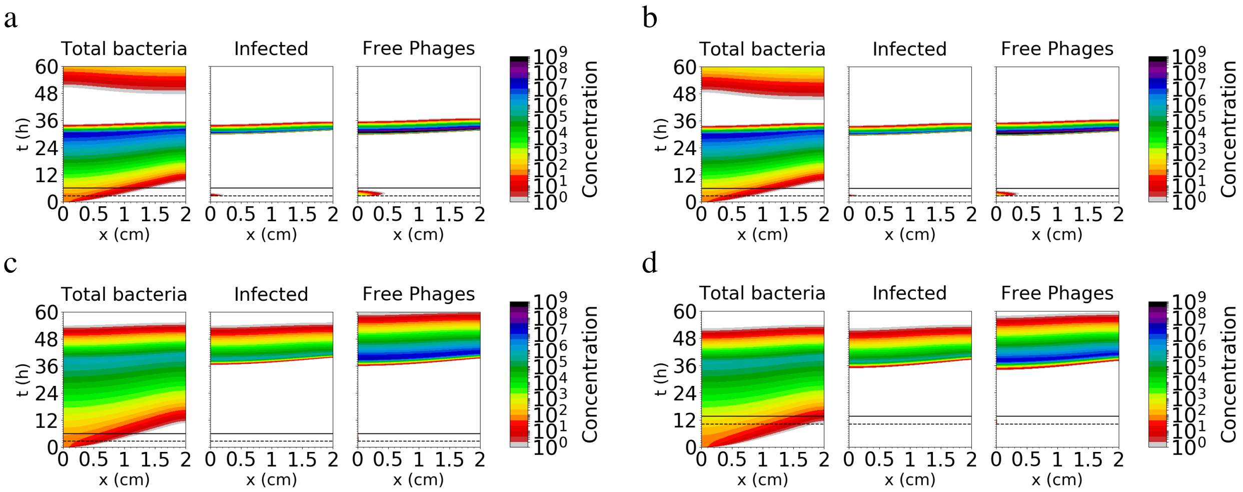

Figure S1. Amounts of total bacteria, infected bacteria and free phages obtained by simulation using the same conditions as in Figure 4, except that washout occurs 3.5 hours after the phage dose.

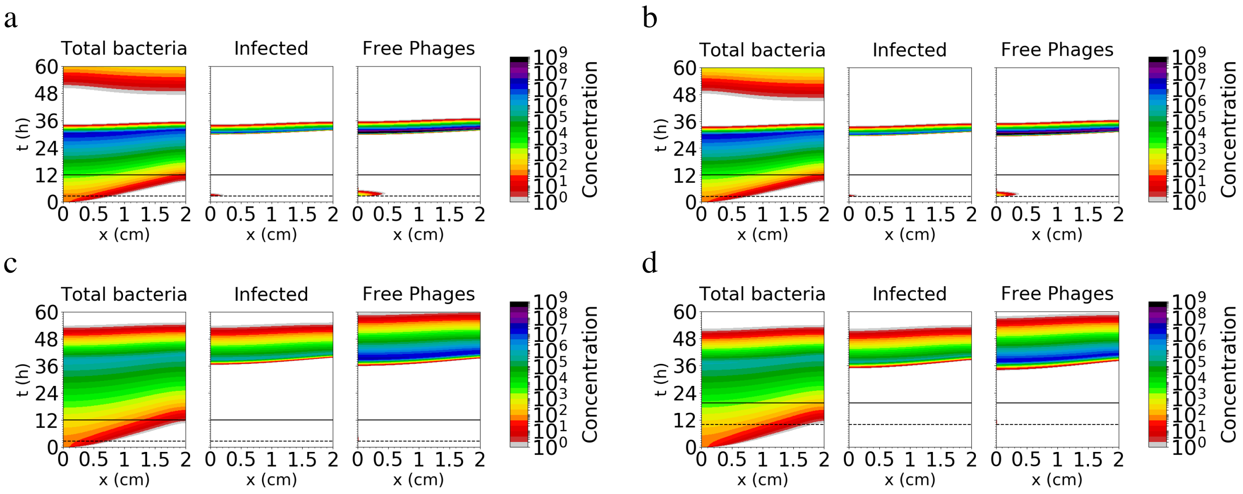

Figure S2. Amounts of total bacteria, infected bacteria and free phages obtained by simulation using the same conditions as in Figure 4, except that washout occurs 9.5 hours after the phage dose.

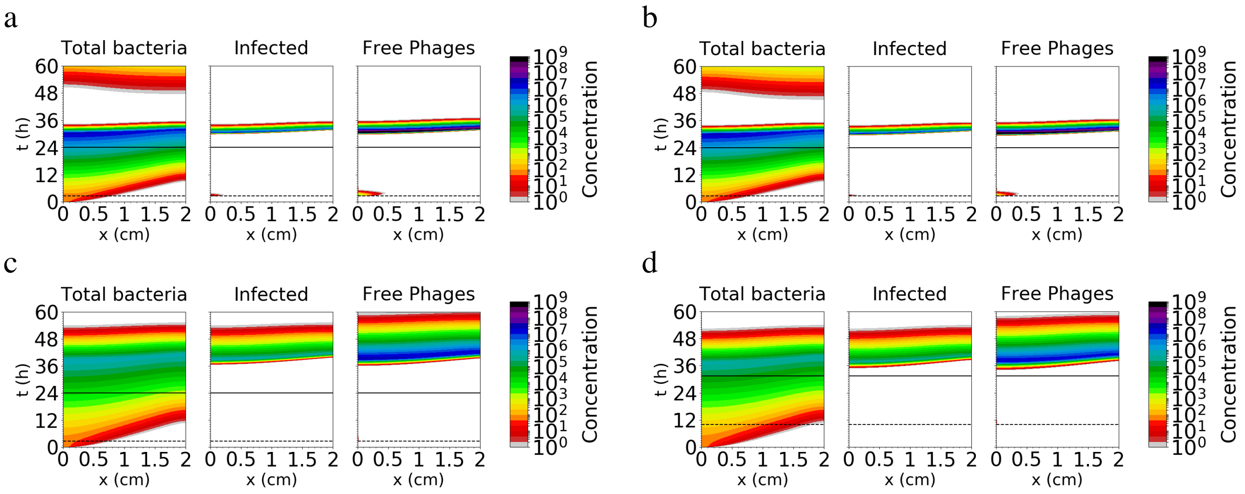

Figure S3. Amounts of total bacteria, infected bacteria and free phages obtained by simulation using the same conditions as in Figure 4, except that washout occurs 21.5 hours after the phage dose.

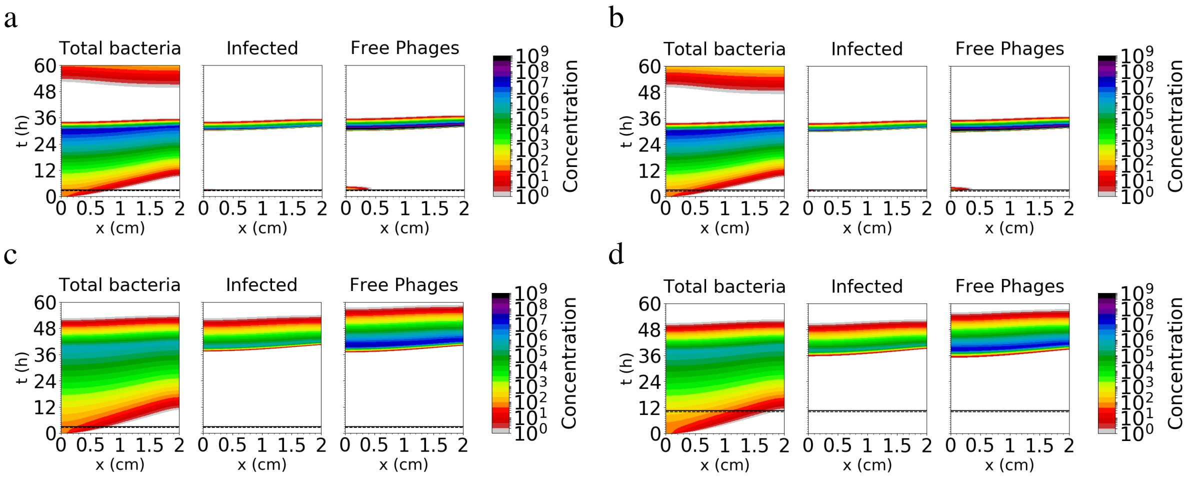

Figure S4. Amounts of total bacteria, infected bacteria and free phages obtained by simulation using the same conditions as in Figure 4, except that uninfected cells replicate with rate *a*/2.

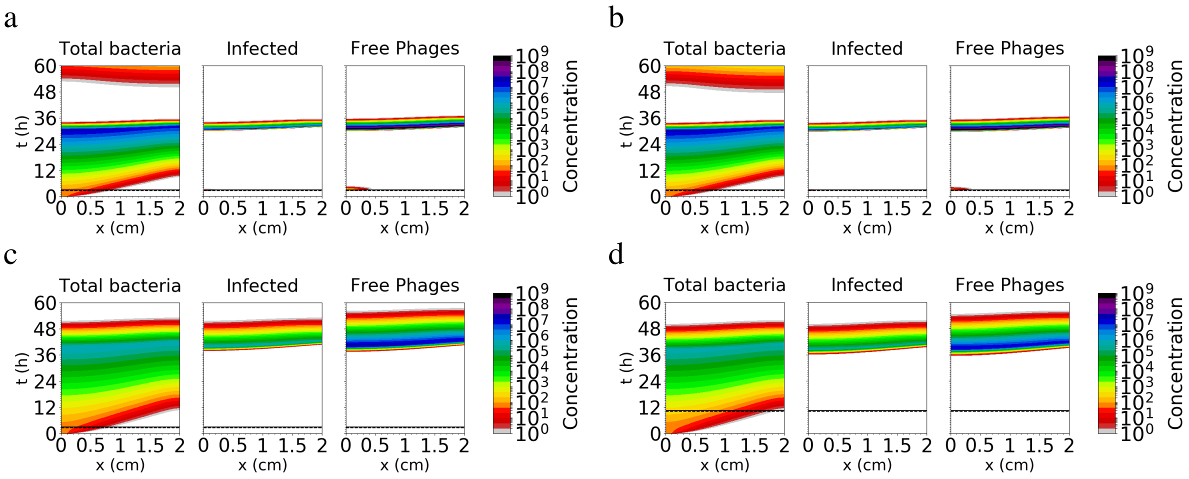

Figure S5. Amounts of total bacteria, infected bacteria and free phages obtained by simulation using the same conditions as in Figure 4, except that uninfected cells replicate with rate $a/10$.
